# A Case Study and Review of the Literature on IgA Nephropathy in Crohn's Disease

**DOI:** 10.1002/ccr3.72418

**Published:** 2026-04-01

**Authors:** Giovanna Fernanda Vazzana, Alessia Romano, Chiara Casuscelli, Alberto La Spada, Domenico Santoro, Roberto Chimenz, Claudio Romano

**Affiliations:** ^1^ Pediatric Gastroenterology and Cystic Fibrosis Unit, Department of Human Pathology in Adulthood and Childhood “G. Barresi” University Hospital “G. Martino” Messina Italy; ^2^ Pediatric Unit, University Tor Vergata Rome Italy; ^3^ Unit of Nephrology and Dialysis, Department of Clinical and Experimental Medicine University of Messina Messina Italy; ^4^ Pediatric Nephrology and Dialysis Unit University Hospital “G. Martino” Messina Italy

**Keywords:** anti‐TNF therapy, Crohn's disease, gut–kidney axis, IgA nephropathy, inflammatory bowel disease, pediatric

## Abstract

IgA nephropathy (IgAN) is the most frequently reported glomerular disease associated with inflammatory bowel disease (IBD), particularly Crohn's disease (CD), although pediatric cases remain rare. We report IgAN in a 16‐year‐old male with CD following intestinal surgery and during long‐term infliximab therapy, with renal impairment occurring independently of bowel disease activity. The patient presented with recurrent macroscopic hematuria, proteinuria, and acute kidney injury despite sustained intestinal remission. Renal biopsy confirmed IgAN (MEST‐C: M0, E0, S1, T0, C1). Treatment with renin–angiotensin system (RAS) blockade and corticosteroids resulted in complete renal remission. Infliximab was discontinued, and a subsequent intestinal flare was successfully treated with ustekinumab. This case highlights the importance of vigilant renal monitoring in pediatric CD, particularly in patients with prior intestinal surgery or long‐term biologic therapy.

## Key Clinical Message (KCM)

IgA nephropathy (IgAN) may develop in pediatric Crohn's disease (CD) independently of intestinal activity, particularly after intestinal surgery and during long‐term biologic therapy. Careful renal monitoring is essential, even in patients with sustained gastrointestinal remission.

## 1 Introduction

Inflammatory bowel diseases (IBD) are chronic, relapsing inflammatory disorders of the gastrointestinal (GI) tract, including Crohn's disease (CD), ulcerative colitis (UC), and IBD‐unclassified (IBD‐U) [1].

Extraintestinal manifestations (EIMs) occur in a significant number of patients, with reported rates ranging from 6% to 47%, and are more common in CD [2]. EIMs can appear before or after the onset of intestinal disease and should be distinguished from extraintestinal complications, which result directly from intestinal inflammation or its metabolic effects [2, 3]. Instead, EIMs reflect inflammatory processes related to IBD development and may share genetic or environmental risk factors. The organs most often affected include the skin (erythema nodosum, pyoderma gangrenosum), joints (peripheral and axial arthropathy), hepatobiliary system (primary sclerosing cholangitis, autoimmune hepatitis), and eyes (uveitis, episcleritis) [4].

Renal involvement is increasingly recognized within the EIMs spectrum, reflecting gut–kidney axis dysregulation driven by chronic inflammation, mucosal immune activation, microbial translocation, and metabolic disturbances [5, 6]. It occurs in 4%–23% of adults and 1%–2% of pediatric patients with IBD [7]. Longitudinal population studies demonstrate that more than 10% of adults with IBD develop chronic kidney disease (CKD) within 10 years of diagnosis, with hazard ratios ranging between 1.2 and 1.9 for both CKD and acute kidney injury (AKI) compared with the general population [8]. The risk is similar for CD and UC and is independent of genetic risk factors for kidney disease [8, 9].

Pediatric and young adult patients exhibit the highest relative risk: up to 19% show reduced eGFR (< 90 mL/min/1.73 m^2^) during follow‐up, particularly in severe or surgically managed disease [10]. These findings support systematic monitoring of kidney function and urinalysis in both pediatric and adult IBD care.

Renal manifestations in IBD (Table 1) are clinically heterogeneous. The most prevalent disorder is nephrolithiasis, which is brought on by the development of uric acid or calcium oxalate stones and, less frequently, calcium phosphate stones with secondary obstructive uropathy. The risk is higher in adults (6%–18%) than in pediatric patients (3%–8%) and in CD than UC. Patients with ileocolonic CD, especially after surgery, are at increased risk of oxalate stone formation due to bile salt malabsorption, hyperoxaluria, dysbiosis, and dehydration [11]. Treatment should focus on increased hydration, urine alkalinization, citrate administration, low‐fat diet, and bile salt sequestrants in cases of steatorrhea or bile salt malabsorption [12].

**TABLE 1:** Renal manifestations in IBD.

Manifestation	Epidemiology	Risk factors	Pathophysiology	Clinical presentation	Management
Glomerulonephritis (IgAN)	Up to 24% of IBD patients in biopsy series; more common in CD	Persistent proteinuria, active IBD, proliferative/sclerotic lesions on biopsy, reduced renal function at onset, anti‐TNF therapy	Aberrant IgA1 overproduction; immune complex deposition in mesangium; intestinal barrier disruption	Microscopic/macroscopic hematuria, proteinuria, hypertension, impaired renal function	RAS blockade for proteinuria/BP control; corticosteroids in high‐risk patients; adjust or withdraw anti‐TNF if suspected drug‐induced; monitor renal function
Nephrolithiasis	Most common Adults 6%–18%, children 3%–8%; CD>UC	Ileocolonic CD, bowel resection, bile salt malabsorption, hyperoxaluria, dehydration	Calcium oxalate or uric acid stones; less frequently calcium phosphate stones.	Renal colic, hematuria, urinary obstruction, or asymptomatic.	Hydration, urine alkalinization, citrate supplementation, low‐fat diet, bile salt sequestrants
Tubulointerstitial nephritis (TIN)	Second most common renal disorder in children with IBD.	5‐Aminosalicylic acid exposure; active IBD	Immune‐mediated or drug‐induced interstitial inflammation	Acute or chronic decline in renal function; may be subclinical.	Drug withdrawal, induce IBD remission.
Proximal tubular dysfunction	Up to 6% of IBD patients	Hypokalaemia, bowel surgery, hyperoxaluria	Impaired reabsorption of filtered solutes in proximal tubules.	Tubular proteinuria; Elevated urinary α1‐ and β2‐microglobulin.	Correction of electrolyte disturbances, treatment of underlying causes.
Amyloidosis (AA)	Rare; CD>UC (10.9% vs. 0.7%) Long‐standing uncontrolled CD	Chronic inflammation, uncontrolled disease	Extracellular deposition of serum amyloid A in glomeruli or renal interstitium.	Proteinuria Nephrotic syndrome Progressive renal insufficiency.	Biologics (e.g., anti‐TNF) may reduce proteinuria and slow CKD progression
Other renal and urological complications	Rare	Chronic inflammation, immunosuppression; penetrating CD	Chronic inflammation, immunosuppression; transmural disease involvement.	Malignancies may be asymptomatic or present with hematuria.	Surveillance; surgical management if indicated.

Tubulointerstitial nephritis (TIN) is the second most common renal disorder affecting children with IBD. Although most cases are frequently associated with drug adverse effects, especially due to exposure to 5‐aminosalicylic acid derivatives, in some cases it is diagnosed prior to initiation of any therapy and is related to IBD activity [11, 12]. Proximal tubular dysfunction has been described in up to 6% of patients with IBD. Low molecular weight proteins, including α1‐ and β2‐microglobulin, are sensitive markers for tubular damage since they are usually filtered by glomeruli and reabsorbed by the proximal tubules. Secondary tubulointerstitial nephritis can result from hypokalemia, particularly in patients who underwent extensive bowel surgery and hyperoxaluria [13].

Amyloidosis is a rare but severe complication of IBD; it is more prevalent in CD compared to UC, with reported incidence respectively of 10.9% and 0.7%, arising predominantly in long‐standing uncontrolled CD. Secondary amyloidosis, the predominant type in patients with IBD, is characterized by extracellular deposition of serum amyloid A in the glomeruli, leading to proteinuria and/or nephrotic syndrome, or in the interstitium, which leads to insufficient kidney function [13]. Treatment aims to reduce serum amyloid A levels by controlling intestinal inflammation in IBD, and infliximab appears to induce disease remission, reduce proteinuria, and slow the progression of chronic kidney disease [12, 13].

Less common complications include urological malignancies related to chronic inflammation and immunosuppression [11, 13]. Penetrating CD can also lead to entero‐vescical fistulas, although rarely, with hematuria or recurrent urinary infections as common symptoms [12].

Importantly, nephrotoxicity is a treatment‐related complication rather than a true EIM [14]. In pediatric IBD, tumor necrosis factor (TNF)‐α inhibitors such as infliximab and adalimumab are central to therapy for moderate‐to‐severe disease [15]; however, IgA nephropathy (IgAN) has emerged as a rare but increasingly recognized adverse effect of anti‐TNF‐α treatment, requiring vigilant renal surveillance in treated patients [16].

Among all renal conditions associated with IBD, glomerular involvement has the most significant prognostic impact. IgAN is the most prevalent primary glomerular disease, with biopsy series reporting rates up to 24% in IBD, significantly higher than in non‐IBD populations [17]. Epidemiologic data support a bidirectional association, showing a 2–3‐fold increased risk of IBD in patients with IgAN as well as increased IgAN risk in individuals with IBD [18]. In pediatric cohorts, cases are uncommon but well documented, including reports that emerge during anti‐TNF‐α therapy [19]. Pathophysiological mechanisms are complex: Immune dysregulation may lead to an inappropriate response to the intestinal microbiota, resulting in disruption of the mucosal barrier and increased translocation of dietary antigens and bacterial toxins [20]. This process triggers the mucosa‐associated lymphoid tissue (MALT) stimulation with subsequent plasma cells activation and production of IgA1 with aberrant glycosylation, formation of circulating immune complexes and deposition in the renal mesangium [20, 21].

Persistent microscopic or macroscopic hematuria, frequently accompanied by proteinuria that occasionally reaches the nephrotic range, are the hallmarks of IgAN [22]. Less common clinical features include hypertension and impaired renal function [22]. Renal manifestations generally occur years after the diagnosis of IBD and are reported more frequently in CD than in ulcerative colitis [17]. Diagnosis requires renal biopsy showing mesangial hypercellularity and segmental glomerulosclerosis, alongside chronic lesions such as interstitial fibrosis and tubular atrophy, particularly in patients with active intestinal disease [22, 23]. Management combines renin–angiotensin system (RAS) blockade for proteinuria and blood pressure control, corticosteroids in selected high‐risk patients, evidenced by persistent proteinuria despite RAS inhibition and in suspected drug‐induced forms, withdrawal or switch of anti‐TNF‐α therapy [24, 25]. The largest multicentre study of IBD‐associated IgA nephropathy, including both pediatric and adult patients, reported a mean follow‐up of 7.2 years [22]. During this period, 16.7% of patients experienced adverse renal outcomes, end‐stage kidney disease (ESKD), or a > 50% decline in eGFR, comparable to primary pediatric IgAN [22]. However, IBD itself is associated with an increased risk of ESKD in IgAN patients, with logistic regression showing an odds ratio of 2.60 and time‐varying Cox regression a hazard ratio of 1.84 [18, 22]. Pediatric IBD‐associated IgAN often presents with active inflammatory lesions and proteinuria, but outcomes are variable, highlighting the heterogeneity of the disease course [26].

Emerging noninvasive biomarkers may enhance the monitoring of pediatric IgAN. Soluble CD89, galactose‐deficient IgA1 (Gd‐IgA1), and IgA immune complexes correlate with proteinuria and proliferative lesions, providing alternatives to repeat biopsies [24]. The IgA/C3 serum ratio and urinary cytokines such as IL‐6 and TGF‐β1 add further prognostic value [24, 27].

Risk factors for progression to CKD include persistent proteinuria, absence of remission, proliferative and sclerotic lesions on biopsy (Oxford E1, S1), and reduced renal function at onset [28].

We describe a rare case of IgAN in a 16‐year‐old male with CD following intestinal surgery and during long‐term infliximab therapy, with renal impairment independent of bowel disease activity. The patient achieved clinical and biochemical remission after combined RAS inhibition, corticosteroid therapy, and proactive withdrawal of infliximab.

## 2 Case History

A 16‐year‐old Caucasian male with a history of CD and a negative family history for kidney disease presented to the pediatric department for the sudden onset of macroscopic hematuria, in the absence of gastrointestinal symptoms. Two weeks before admission, he had experienced an upper respiratory tract infection. CD had been diagnosed 6 years earlier, with ileocolonic involvement and perianal disease (Paris classification: A1b, L3, B2, G0, P). Two years after diagnosis, he underwent perianal surgery for a complex fistula, followed by ileocecal resection due to stenotic disease. Postoperatively, he received maintenance therapy with infliximab, administered at a dose of 5 mg per kilogram every 8 weeks, which he continued for several years, during which clinical remission of intestinal disease was maintained.

At admission, the patient appeared in good general condition, without edema or hypertension. Urinary sediment examination with phase‐contrast microscopy revealed hematuria with dysmorphic red blood cells (acanthocytes), and chemical–physical urine test showed proteinuria of 100 mg/dL (reference range, 0–10 mg/dL) with a negative urine culture. Laboratory tests revealed AKI, with elevated serum creatinine (1.7 mg/dL; reference range 0.5–1.2 mg/dL) and blood urea nitrogen (66 mg/dL; reference range 10–50 mg/dL). eGFR was 69 mL/min/1.73 m^2^, calculated using the Chronic Kidney Disease Epidemiology Collaboration (CKD‐EPI) equation. Serum IgA levels were elevated (516 mg/dL; reference range 61–301 mg/dL), whereas complete blood count, IgG, C‐reactive protein, erythrocyte sedimentation rate, fecal calprotectin, and complement levels (C3 and C4) were within normal limits. Serum albumin was normal. Autoimmune serologies (extractable nuclear antigen, anti–double‐stranded DNA, antineutrophil cytoplasmic antibodies, antinuclear antibodies, anti–glomerular basement membrane antibodies) were negative.

During the subsequent months, he experienced two additional episodes of macroscopic hematuria, both coinciding with minor respiratory infections.

## 3 Differential Diagnosis, Investigations, and Treatment

Given the recurrent episodes of hematuria, persistent proteinuria, and elevated IgA levels, IgAN was suspected. Infliximab therapy was temporarily discontinued during the diagnostic evaluation, although there were no definitive clinical data to implicate this therapy as causal.

Differential diagnosis included other causes of hematuria and proteinuria in pediatric patients with IBD: drug‐induced nephrotoxicity, postinfectious glomerulonephritis, secondary amyloidosis, tubulointerstitial nephritis, and urological complications. The absence of urinary infection, normal complement levels, negative autoimmune serologies, and persistent proteinuria favored IgAN.

**FIGURE 1:**
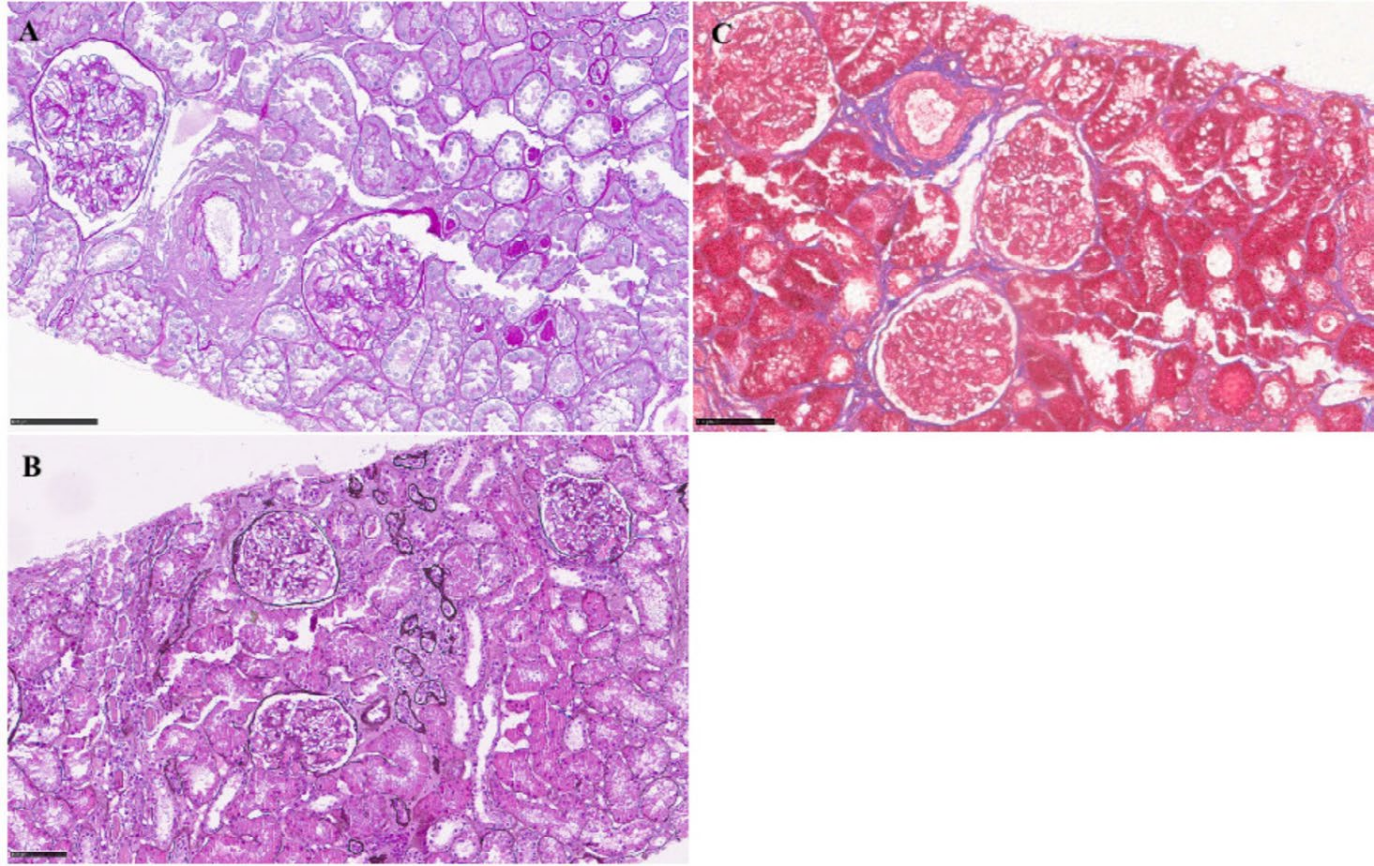
Light microscopy: (A) PAS staining showing increase in mesangial cellularity (< 50% of glomeruli) with crescents in two glomeruli. (B) PASM staining demonstrating preserved glomerular basement membranes. (C) Masson's trichrome staining revealing mild interstitial fibrosis and tubular atrophy. Scale bar: 100 μm.

**FIGURE 2:**
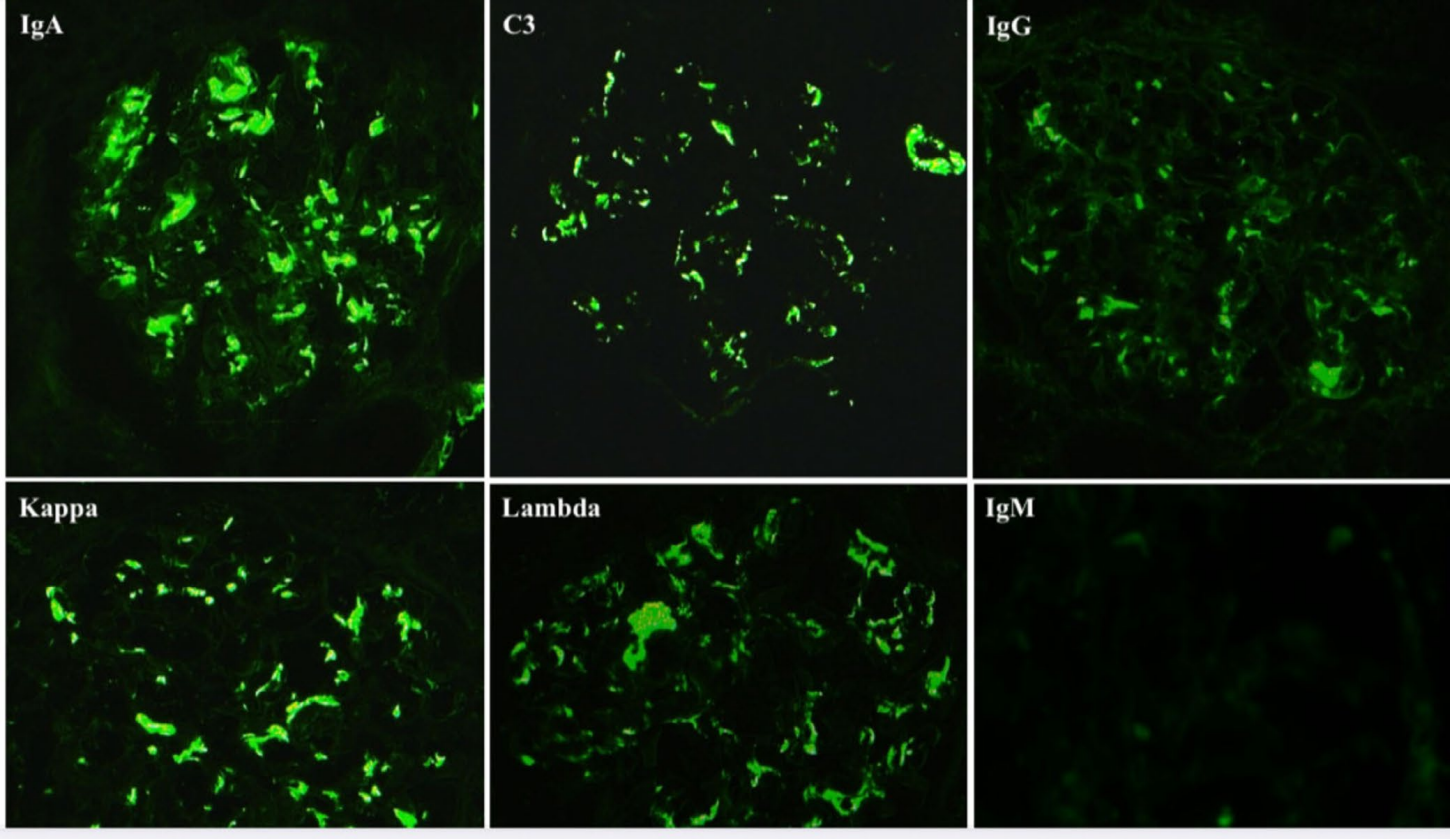
Immunofluorescence microscopy of renal biopsy showing granular mesangial deposition of IgA (+++), C3 (++), weak IgG staining (+), and IgM staining was negative. Kappa (++) and lambda (+++) light chains show mesangial positivity with a polyclonal pattern. Magnification 20×.

A percutaneous kidney biopsy was performed. Histopathology confirmed IgAN (MEST‐C score: M0, E0, S1, T0, C1). Light microscopy revealed mesangial proliferation in fewer than 50% of glomeruli and showed two glomeruli with crescent formation; there was approximately 10% mild tubular atrophy and interstitial fibrosis, accompanied by a slight lymphomonocytic infiltrate. Periodic acid Schiff (PAS) staining highlighted mesangial matrix expansion and hypercellularity. Periodic acid methenamine silver (PASM) staining demonstrated preserved glomerular basement membranes without spikes or double contours. Masson's trichrome staining revealed mild interstitial fibrosis and tubular atrophy involving approximately 10% of the cortical area (Figure 1). Immunofluorescence studies revealed granular mesangial deposition of IgA (+++), accompanied by C3 (++), with weak IgG staining (+). IgM staining was negative. Kappa (++) and lambda (+++) light chains showed mesangial positivity with a polyclonal pattern (Figure 2).

Treatment was started with RAS inhibitors and reduced‐dose corticosteroids according to the Pozzi protocol (methylprednisolone 500 mg/day intravenously for three consecutive days in months 1, 3, and 5, followed by oral prednisone 0.5 mg/kg on alternate days for 6 months), resulting in prompt clinical and laboratory remission and normalization of renal function [29].

## 4 Conclusion and Results (Outcome and Follow‐Up)

At 12‐month follow‐up, renal function improved, with a serum creatinine level of 1.0 mg/dL (reference range 0.5–1.2 mg/dL), an eGFR of 111.9 mL/min/1.73 m^2^ and a blood urea nitrogen concentration of 36 mg/dL (reference range 10–50 mg/dL). No further episodes of macroscopic hematuria were observed.

From a gastrointestinal standpoint, the patient remained in clinical remission with a wPCDAI < 10, fecal calprotectin < 100 μg/g, and a normal magnetic resonance enterography during the episodes of hematuria. However, two months after infliximab withdrawal, he developed a flare of CD characterized by abdominal pain and diarrhea. Endoscopic reevaluation showed active disease (Rutgeerts score i2). Therapy with ustekinumab, a fully human IgG1κ monoclonal antibody targeting IL‐12/23, was initiated, with a good therapeutic response (see Table 2. for clinical summary of the reported case). Written informed consent for publication of this case report was obtained from the patient's parents.

**TABLE 2:** Clinical summary of the reported case.

Category	Findings
Demographics	16‐year‐old Caucasian male; no family history of renal disease
CD phenotype	Ileocolonic disease with perianal involvement (Paris A1b, L3, B2, G0, P)
Age at CD diagnosis	10 years
Surgical history	Perianal surgery for complex fistula and ileocecal resection (~15‐cm terminal ileum + cecum) at age 12
IBD therapy	Long‐term infliximab (4 years) with sustained intestinal remission
Onset of renal involvement	Macroscopic hematuria at 16 years, following upper respiratory infection; absence of active IBD symptoms
Renal laboratory findings	Proteinuria (100 mg/dL); dysmorphic hematuria; elevated serum IgA; AKI (creatinine 1.7 mg/dL; eGFR 69 mL/min/1.73 m^2^)
Kidney biopsy	IgAN; MEST‐C: M0, E0, S1, T0, C1; mild mesangial proliferation; two small crescents
Management	RAS blockade + Pozzi corticosteroid protocol; temporary infliximab discontinuation
Outcome	Renal function improved within 12 months; later CD flare, controlled after switch to ustekinumab

## 5 Discussion

IgAN is the most frequently reported form of primary glomerulonephritis in patients with IBD [17]. Epidemiological data demonstrate that individuals with CD have a higher risk of developing IgAN compared with both the general population and those with UC [30]. Part of this association may derive from shared genetic susceptibility. In particular, HLA‐DR1–positive patients have demonstrated increased vulnerability to both CD and IgAN, supporting the concept that antigen presentation pathways relevant to intestinal inflammation may also drive aberrant IgA immune responses [31]. Additionally, emerging studies suggest that HLA‐DQA1*05, a genotype associated with heightened immunogenicity to anti‐TNF–α agents, may be a potential modifier of renal immune injury in susceptible individuals, although a direct causal link to IgAN development has not yet been established [32].

The biological connection between intestinal inflammation and renal immune complex disease is increasingly explained through the gut–kidney axis, a bidirectional network linking mucosal barrier integrity, microbiota homeostasis, and systemic immune regulation [5, 33]. In CD, chronic intestinal inflammation, dysbiosis, and impaired mucosal barrier function favor translocation of microbial antigens and persistent activation of the gut‐associated lymphoid tissue [34]. In predisposed subjects, excessive production of Gd‐IgA1, which is more prone to forming circulating immune complexes, has been described [20, 33]. When these complexes deposit within the renal mesangium, they trigger complement activation and glomerular inflammation characteristic of IgAN [34, 35].

Moreover, IBD‐related dysbiosis increases systemic cytokines and uremic toxins such as indoxyl sulfate and p‐cresyl sulfate, amplifying tubular and glomerular injury [35, 36]. This immunological interplay supports the well‐established association of IBD not only with IgAN but also with other glomerular diseases including focal segmental glomerulosclerosis [20, 36].

Our case is particularly notable because IgAN developed after ileocecal resection and during prolonged remission on biologic therapy. This supports the hypothesis that surgical disruption of mucosal architecture and immune homeostasis can amplify antigen translocation and immunologic dysregulation along the gut–kidney axis [15, 20, 35]. Ileocecal resection may contribute to IgA nephropathy by disrupting mucosal immune homeostasis and altering intestinal permeability and microbiota composition. Loss of Peyer's patch–mediated tolerance may lead to exaggerated immune responses to luminal antigens and microbial products, promoting systemic immune activation [33, 37]. These alterations favor the production of aberrantly glycosylated polymeric IgA1 and its deposition in the kidney [37]. Patients with IgA nephropathy exhibit increased circulating gut‐homing (CCR9+ β7 integrin+) regulatory and memory B cells, together with elevated IgA+ plasmablasts that preferentially migrate to extraintestinal sites [33, 37]. Moreover, the gut microbiome is increasingly recognized as a direct modulator of IgA1 structure: microbial glycosidases can induce deglycosylation of IgA1, generating neo‐antigenic epitopes that drive the formation of pathogenic IgA–IgG immune complexes [35, 37]. Notably, mucin‐degrading bacterial species such as *Akkermansia muciniphila* have been implicated in these processes, reinforcing the concept of a disturbed gut–kidney axis in genetically susceptible individuals [34, 37].

In our case, the absence of gastrointestinal symptoms at the time of renal involvement suggests that renal injury may progress independently of clinical intestinal disease, driven by subclinical mucosal inflammation and postsurgical immune alterations.

Although several case reports have suggested a temporal association between anti‐TNF‐α therapies, particularly infliximab, and the onset or relapse of IgAN, causality remains uncertain [38]. Proposed mechanisms include immunogenicity against chimeric antibodies, the generation of autoantibodies recognizing aberrant IgA1, and a shift toward humoral immune responses secondary to TNF‐α blockade [39, 40]. In genetically predisposed patients, these alterations may facilitate the formation and renal deposition of immune complexes [32, 39]. However, current evidence is limited, and it is not possible to establish a definitive causal relationship. Anti‐TNF‐α therapy may act as a cofactor, a risk factor, or potentially a driver in susceptible individuals, particularly when other risk factors, such as intestinal surgery, are present [40, 41]. In the present case, a temporal association with biologic therapy was observed, but it remains difficult to attribute IgAN solely to intestinal resection or to any single factor. This underscores the complexity of immune interactions in pediatric CD and highlights the need for further prospective and mechanistic studies to clarify the contribution of surgery, chronic inflammation, and biologic therapy to renal immune pathology.

When IgAN is diagnosed during anti‐TNF therapy, the decision to continue, switch, or discontinue biologic therapy must be individualized, weighing renal and intestinal disease severity [38, 41]. Until more robust data are available, careful renal monitoring in pediatric patients, especially those undergoing intestinal surgery or receiving long‐term anti‐TNF‐α therapy, remains essential, even in the absence of active gastrointestinal disease.

This case underscores that IgAN may occur in pediatric CD even during sustained intestinal remission, highlighting that renal involvement can develop independently of overt gastrointestinal activity (see Table 3. for pediatric cases of IgAN in CD).

**TABLE 3:** IgAN in CD: Pediatric cases.

Reference/year	Age (years)	Sex	CD phenotype	Intestinal resection	CD therapy at IgAN onset	Time to IgAN onset	IgAN treatment	CD activity at IgAN onset	Outcome
Dabadie A. et al. 1996 [42]	12	F	Ileocecal stenotic disease	Yes	Surgical and nutritional therapy	1 year after CD diagnosis	Clinical and laboratory renal monitoring	Yes	Persisten micro hematuria
McCallum D. et al. 1997 [43]	11	F	Not specified	Not reported		4 years before CD	Clinical and laboratory renal monitoring		Persistent microhematuria
Takemura T. et al. 2002 [44]	10	M	Not specified	Not reported		3 years before CD diagnosis	Prednisolone, cyclophosphamide, warfarin, and ACE inhibitor		IgAN flare at CD diagnosis, followed by remission after steroid therapy
Graziano et al. 2022 [19]	11	M	Ileal and jejunal	Not reported	Anti‐TNF‐α (adalimumab)	2 years after CD diagnosis	Steroids and ACE inhibitor (after stopping adalimumab)	Remission	Remission
Tang J. Et al. 2025 [45]	11	F	Ileocolonic	Not reported	Anti‐TNF‐α (Infliximab)	4 years after CD diagnosis and 1 year after Infliximab starting	Clinical and laboratory renal monitoring	Remission	Persistent microhematuria
Sugino et al. 2025 [46]	10	M	Ileocolonic	Not reported	Infliximab	2 years after CD diagnosis	Upadacitinib+infliximab	Remission	Remission

The temporal link with ileal resection and long‐term anti‐TNF‐α therapy suggests that both surgical disruption of the gut–kidney axis and biologic exposure could contribute to disease onset, although neither can be considered a definitive cause. These observations emphasize the importance of systematic renal monitoring in pediatric patients, particularly following intestinal surgery or prolonged biologic therapy.

Further prospective and pathophysiological studies are warranted to elucidate the interplay among gut–kidney axis disruption, immunomodulatory therapies, and genetic susceptibility, as well as to identify biomarkers enabling early detection and targeted prevention.

## Author Contributions

**Giovanna Fernanda Vazzana:** writing – review and editing. **Alessia Romano:** writing – review and editing. **Chiara Casuscelli:** visualization. **Domenico Santoro:** visualization. **Roberto Chimenz:** visualization. **Claudio Romano:** supervision. **Alberto La Spada:** visualization.

## Funding

The authors have nothing to report.

## Ethics Statement

As a single case report with the patient's signed consent, no other ethical review was required.

## Consent

Written informed consent was obtained from the patient for publication of this case report and accompanying images, in accordance with journal requirements.

## Conflicts of Interest

The authors declare no conflicts of interest.

## Data Availability

All data generated or analyzed during this case report are included in this published article.
